# Dual-nozzle microfluidic droplet generator

**DOI:** 10.1186/s40580-018-0145-2

**Published:** 2018-05-08

**Authors:** Ji Wook Choi, Jong Min Lee, Tae Hyun Kim, Jang Ho Ha, Christian D. Ahrberg, Bong Geun Chung

**Affiliations:** 1 Department of Mechanical Engineering, Sogang University, Seoul, 04107 Republic of Korea; 2 Research Center, Sogang University, Seoul, 04107 Republic of Korea

**Keywords:** Dual-nozzle, Droplet, Dispensing, Microfluidic device

## Abstract

The droplet-generating microfluidics has become an important technique for a variety of applications ranging from single cell analysis to nanoparticle synthesis. Although there are a large number of methods for generating and experimenting with droplets on microfluidic devices, the dispensing of droplets from these microfluidic devices is a challenge due to aggregation and merging of droplets at the interface of microfluidic devices. Here, we present a microfluidic dual-nozzle device for the generation and dispensing of uniform-sized droplets. The first nozzle of the microfluidic device is used for the generation of the droplets, while the second nozzle can accelerate the droplets and increase the spacing between them, allowing for facile dispensing of droplets. Computational fluid dynamic simulations were conducted to optimize the design parameters of the microfluidic device.

**Electronic supplementary material:**

The online version of this article (10.1186/s40580-018-0145-2) contains supplementary material, which is available to authorized users.

## Introduction

The development of microfluidics and micro total analysis systems (µTAS) [1] has led to a paradigm shift in many research areas. Microfluidics allows the precise handling of small volumes of liquids, while maintaining a high control over mass and thermal transport, as well as fast response times at low cost and automation [2]. Two options exist for the operation of a microfluidic device, either continuous or segmented flow. While in continuous flow only one phase is used [3, 4], segmented flow breaks up the flow using two or more different phases [5]. Despite the higher complexity, the segmented flows possess a number of advantages over continuous flows. Typically, droplets provide faster mass and thermal transfer, while preventing boundary effects, such as axial dispersion. Furthermore, they provide small, reproducible volumes, can be manipulated independently, and serve as individual units for reactions [6]. Due to their high homogeneity and fast mass transfer, they are commonly used for the controlled synthesis of nanoparticles [7, 8]. Other applications can be found in the creation of artificial cells [9], the analysis of single cells [10], or in digital polymerase chain reaction [11]. For all of these applications, the generation of stable and monodispersed droplets is necessary.

In microfluidics, droplets can be made either following an active or a passive method. In active methods, droplets are generated by applying an external force. This can be done either by applying a direct or alternating current. In systems consisting of one conducting and one insulating phase, charges accumulate on the interface due to electrochemical reactions. The resulting electrical field force results in the formation of droplets [12]. Alternatively, a force can be created through thermal expansion of one of the two phases, as can be done by localized laser irradiation [13, 14]. Lastly, droplets can be generated by active methods utilizing active valves or pneumatically actuated membranes [15, 16]. In passive method, pressure-driven flows of the dispersed and continuous phase meet at a microchannel junction. The characteristics of the junction determine the interface deformation and the formation of droplets. One, infrequently used, option is to arrange both streams in coaxial microchannels. The dispersed phase is introduced in the central channel, while the continuous phase flows through outer channels [17, 18]. Similarly, flow-focusing geometries use a central flow of the dispersed phase and outer flows of segmented phase. In contrast to coaxial microchannels, the flows pass a contraction region after which the central flow breaks up into droplets [19, 20]. The most popular method for passive droplet generation is the cross-flow method. Here, the flow of the continuous phase is partially blocked by a flow of the dispersed phase coming from a secondary channel. Through this, a shear gradient develops, the dispersed phase elongates and eventually breaks into droplets [21–23]. Some applications, such as filling of nanowells [24] or production of micro-lenses [25] require the dispensing of the generated droplets. Previously, this has been obtained by generating droplets using a pinched flow channel, followed by injecting the droplets into a stream of a carrier gas for analysis in ion coupled mass spectroscopy (ICPMS) [26]. Other groups have been able to achieve dispensing by either precise timing control of the dispensing process [27], or by the use of an active piezo-electric droplet generator [28]. Through the use of an active droplet generation method, the issue of droplet aggregation and merging can be prevented. However, this reduces the throughput of the microfluidic device and adds complexity to the system.

Here, we show a novel method of droplet dispensing using a dual-nozzle microfluidic setup. While the first nozzle is used for the generation of droplets, the second nozzle is used for the acceleration of the generated droplets and to increase the spacing between them. Through this droplets can be dispensed at a high frequency without the issue of aggregation and merging at the device outlet. A computational fluid dynamic (CFD) model was created before experiments to optimize the design of microfluidic devices.

## Methods

### Computational model of the microfluidic device

Prior to experiments, the performance of the dual-nozzle droplet-generating microfluidic device was simulated using CFD model. For this purpose, the two phase flow with level set function of COMSOL (5.1, COMSOL Inc., USA) was used as previously suggested [29]. The governing equations for the simulation are the Navier–Stokes equation and the continuity equation for the conservation of momentum and mass:

$$\uprho\frac{{\partial {\mathbf{v}}}}{\partial t} +\uprho\left( {{\mathbf{v}} \cdot \nabla } \right){\mathbf{v}} = \nabla \cdot \left[ { - p{\mathbf{I}} + \mu \left( {\nabla {\mathbf{v}} + \left( {\nabla {\mathbf{v}}} \right)^{T} } \right)} \right] + {\mathbf{F}}_{{{\mathbf{st}}}}$$

$$\nabla \cdot {\mathbf{v}} = 0$$

where **v**, *p*, and **F**_**st**_ are the velocity vector, pressure, and the surface tension, respectively. The density and dynamic viscosity is denoted by ρ and *μ*, respectively. The position of the phase interface can be tracked using the level set function as a transportation equation:

$$\frac{\partial \phi }{\partial t} + {\mathbf{v}} \cdot \nabla \phi = \gamma \nabla \cdot \left( { - \phi \left( {1 - \phi } \right)\frac{\nabla \phi }{{\left| {\nabla \phi } \right|}} + \varepsilon \nabla \phi } \right)$$

where *ϕ* is the level set function, and *γ* and *ɛ* are numerical stabilization parameters. The following equations were used for the Multiphysics coupling of density and viscosity:

$$\uprho =\uprho_{1} + \left( {\uprho_{2} -\uprho_{1} } \right)\phi$$

$$\upmu =\upmu_{1} + \left( {\upmu_{2} -\upmu_{1} } \right)\phi$$

For the simulations, a value of *ρ*_1_ = 800 kg/m^3^ and dynamic viscosity of *μ*_1_ = 0.01 Pa s was used. For water, the values were *ρ*_2_ = 1000 kg/m^3^ and *μ*_2_ = 0.001 Pa s, respectively. Furthermore, all fluids were assumed to be incompressible, homogenous Newtonian fluids. A model of the microfluidic droplet dispensing device was constructed based on the AutoCAD drawing used for device fabrication. The walls were defined as wetted boundaries with a contact angle of 120° for the water phase and no pressure was set at the outlet of the microfluidic device.

### Fabrication of the dual-nozzle microfluidic device

A microfluidic dual-nozzle device consisting of two inlets for each nozzle was designed using AutoCAD (Autodesk, USA) and printed onto photomasks. All inlet channels were designed with a width of 70 µm with the exception of the water inlet in the first nozzle which had a width of 100 µm. The design from the masks was transferred to silicon wafers (Wangxing Silicon-Peak Electronics, China) using a standard soft-lithography process as shown previously [30]. Briefly, silicon wafers are cleaned using a wafer washing system and afterwards dried for 5 min at 200 °C on a hotplate. 5 mL of SU-8 50 photoresist (Microchem Corp., USA) was spin-coated onto the silicon wafers at 3000 rpm for 60 s, resulting in a 40 µm photoresist layer. The spin-coated wafer was soft-baked at 65 °C for 5 min and afterwards further heat treated at 95 °C for 15 min on a hotplate to evaporate the solvent. After UV-exposure for 10 s at an intensity of 20 mW/cm^2^, the wafers were baked at 65 °C for 1 min, followed by heat treatment at 95 °C for 4 min on a hotplate. The silicon masters were developed using SU-8 developer (Microchem Corp., USA) and dried with air. Poly(dimethylsiloxane) (PDMS, Dow Corning, USA) was poured onto the silicon wafers. After curing in an oven at 80 °C, the PDMS was peeled off from the silicon wafer and was subsequently bonded into glass slides using oxygen plasma.

### Droplet dispensing experiments

Syringe pumps (PHD 2000, Harvard Apparatus, USA) were connected to the four inlets of the microfluidic device using tygon tubing (Sigma Aldrich, USA) to conduct droplet dispensing experiments. For experiments, de-ionized water (DI water) was used as the continuous phase and mineral oil (M5904, Sigma Aldrich, USA) as the dispersed phase. For experiments, all flow rates were systematically varied between 10 and 50 µL/min in increments of 10 µL/min, in accordance with the values previously used for numerical simulations. Images of the resulting droplets were captured using an inverted microscope (Olympus IX73, Japan) and were also analyzed using Image J (National Institute of Health, USA) regarding their droplet diameter and the distance between droplets.

## Results and discussion

### Fabrication of dual-nozzle microfluidic device

**Fig. 1:**
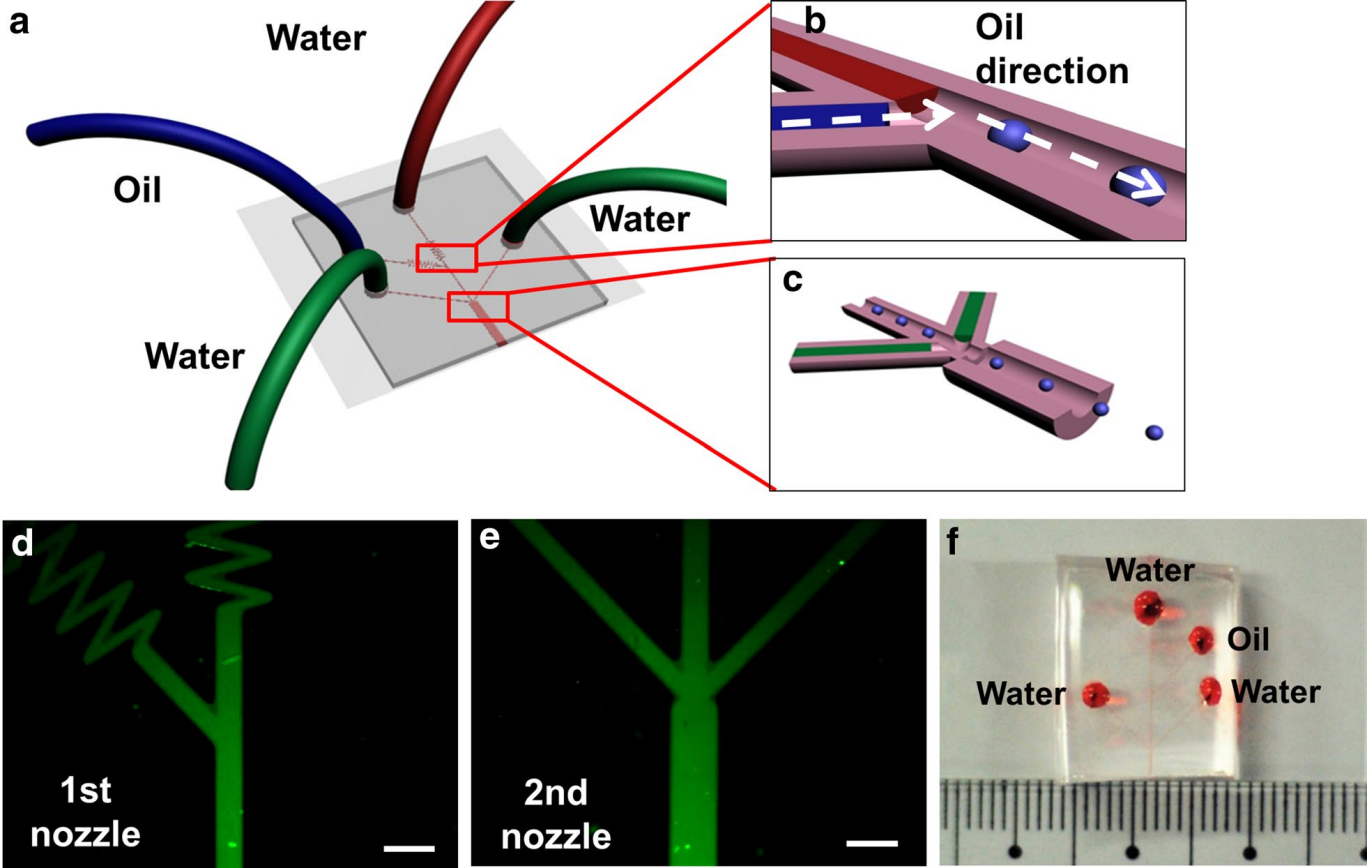
Design and fabrication of the microfluidic dual-nozzle device. Schematic of the dual-nozzle device (**a**), magnified schematic of the first (**b**), and second nozzle (**c**). Microscope images of the fabricated first (**d**), and second nozzle (**e**). For illustration purposes, the channels are filled with fluorescein solution. Scale bars are 200 µm. Photograph of the fabricated PDMS device, for illustration purposes the channels are filled with red dye (**f**)

The dual-nozzle microfluidic device consisting of three water inlets and one oil inlet combined into two nozzles (Fig. 1). The first nozzle which formed a Y-arrangement (Fig. 1b) was used for the generation of mineral oil droplets. In the second nozzle area, the distance between the formed mineral oil droplets could be adjusted through the injection of further water (Fig. 1c). Overall, the microfluidic device has an area of less than 2.5 cm^2^, making it easily to integrate into various applications (Fig. 1f).

### Computational model

**Fig. 2:**
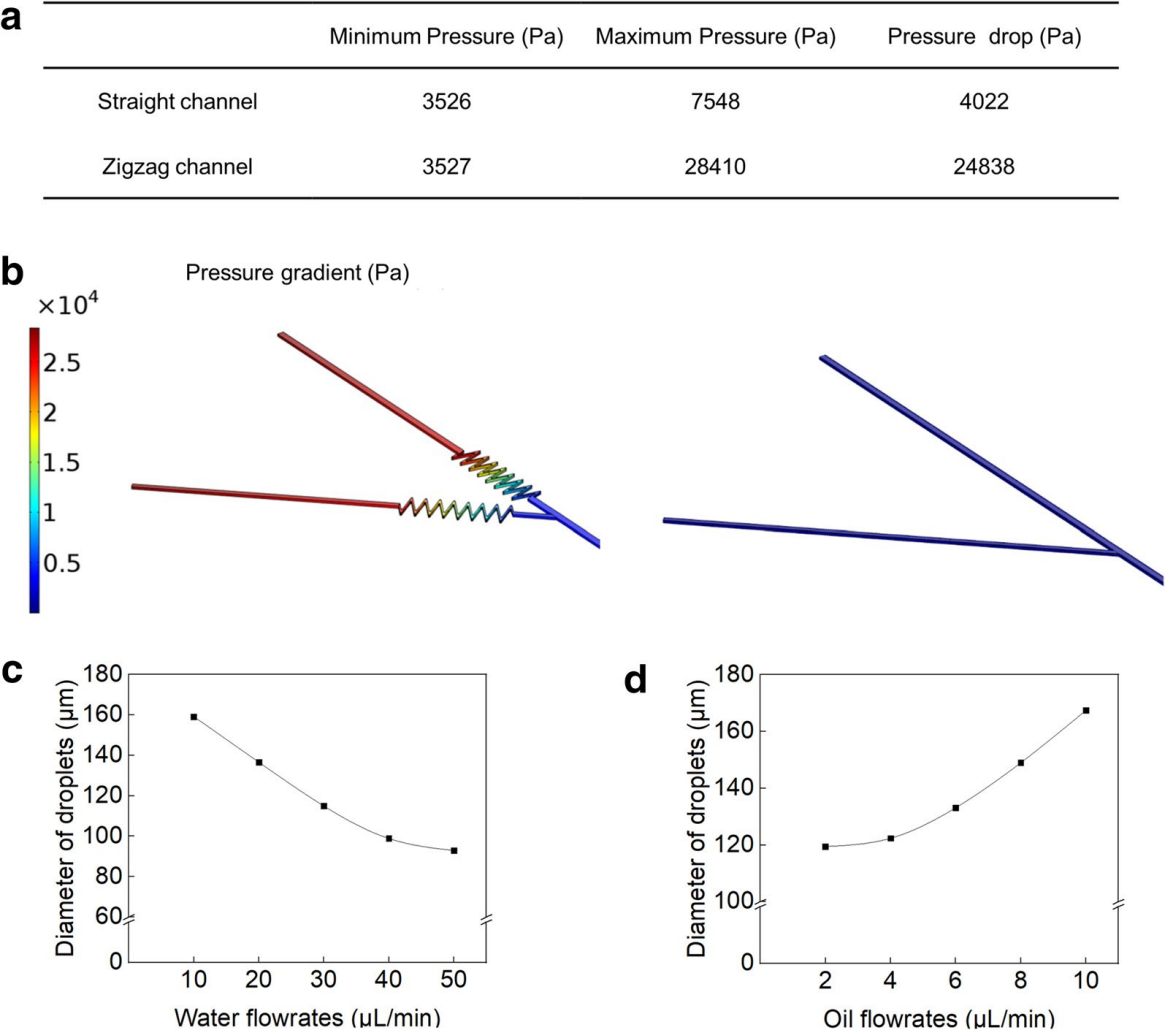
CFD simulation of the first nozzle of the microfluidic device. Table comparing the pressure drop from the inlet to the first nozzle for a straight and zigzag channel (**a**). Snapshot of pressure distribution in the first nozzle for the cases in which a zigzag channel or a straight channel is used (**b**). Simulation of droplet diameter for cases in which the water flow rate (**c**), or oil flow rate (**d**) is varied and the other flowrate remains constant

Prior to experiments, CFD simulations were carried out to optimize the device design for droplet generation and dispensing applications. A first concern was the generation of back flow in the device, especially through the generation of back pressure by the second nozzle. Hence, two different designs for the first nozzle were simulated. A first design with straight connections between the first nozzle and the corresponding inlets, and a second design with zigzag channels between the inlets and the first nozzle were tested (Fig. 2). The simulations predict that through the introduction of the zigzag channel, the pressure drop between the device inlet and the first nozzle increases by a factor of 6 (Fig. 2a, b), making the device more robust to back pressure. While Hagen–Poiseuille predicts only an increase of a factor two through the increase of channel length, the sharp corners of the channel cause a higher robustness to back pressure [31, 32]. Although the addition of the zigzag channel increases the size of the device, the increase in size is smaller than would be required by just increasing the length of the channels. Next, the generation of droplets in the first nozzle was simulated, as well as the how the droplet volume can be controlled by the water and oil flow rates (Fig. 2c, d). When the water flow rate was increased and the oil flow rate remained constant, the frequency of oil droplet generation was increased. This caused a decrease of the diameter of generated oil droplets from 160 µm at a water flow rate of 10 µL/min to 90 µm at 50 µL/min (Fig. 2c). In contrast, when the oil flow rate is increased and the water flow rate kept constant, simulations predict and increase in the oil droplet size (Fig. 2d).

### Droplet generation in a dual-nozzle microfluidic device

**Fig. 3:**
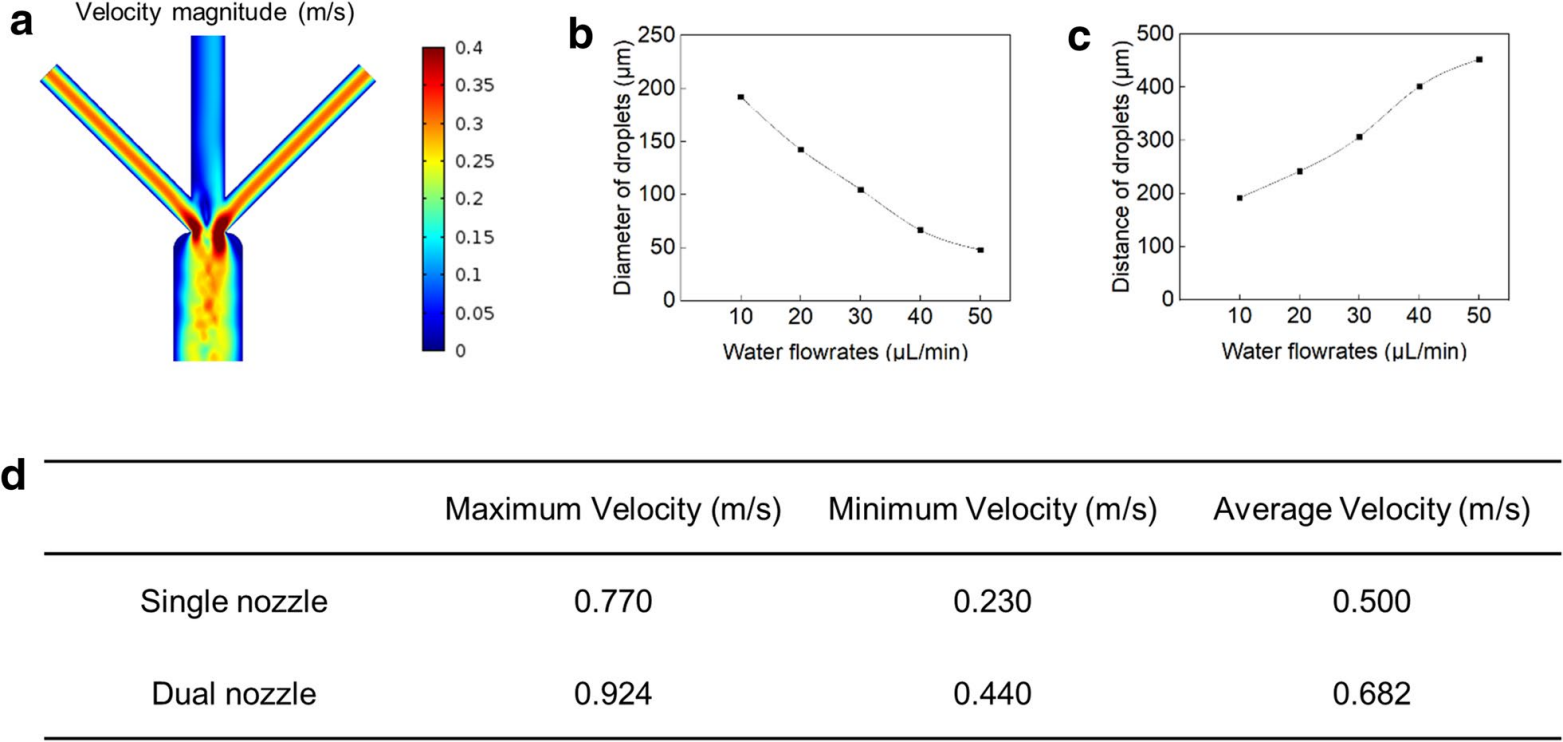
Simulation of the second nozzle of the microfluidic device. Snapshot of the fluid velocity distribution around the second nozzle of the microfluidic device (**a**). Graphs of diameter of droplets generated at second nozzle against water flowrate (**b**). Graph of simulated distance of droplets after second nozzle as a function of water flow rate (**c**). Table of flow velocities for a single and dual-nozzle microfluidic device (**d**)

**Fig. 4:**
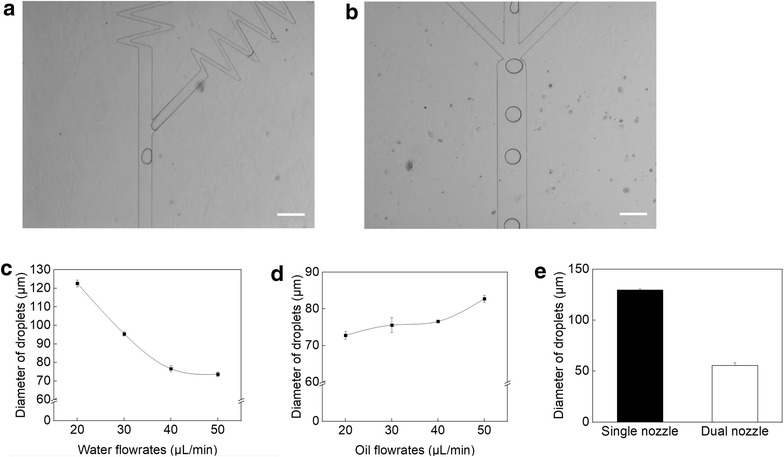
Droplet generation and spacing using dual-nozzle microfluidic device. Microscope image of droplet generation at first nozzle (**a**) and control of droplet distance at second nozzle (**b**). Scale bars are 200 µm. Graphs of diameter of droplets generated at first nozzle against water flowrate (**c**), and oil flowrate (**d**), and bar diagram of dispensed droplet diameter at device outlet using a single nozzle and the dual-nozzle setup (**e**)

The droplet dispensing device had an integrated second nozzle to adjust the distance between the droplets generated in the first nozzle. Furthermore, this second nozzle should prevent droplet aggregation and merging. As for the first nozzle, the behavior of the second nozzle was also simulated using CFD models prior to experiments (Fig. 3). Through the injection of additional water, the distance between the individual droplets could be increased due to the velocity difference before and after the second nozzle (Fig. 3a, d). However, the increase in flow rate at the second nozzle causes a backpressure influencing the first nozzle. Through this, simulation predicts a decrease in droplet size generated by the first nozzle (Fig. 3b). The simulation predicts an almost linear relationship between the distance of the droplets and the flow rate of the additional water injection (Fig. 3c). Through the adjustment of the droplet distances, the agglomeration and merging of droplets at the outlet of the microfluidic device could be prevented and the dispensing of droplets achieved in simulations. After the simulation of the droplet dispenser, experiments were conducted using the previously fabricated PDMS device (Fig. 4). For the experiments, water and oil flow rates equivalent to the flow rates in simulations were used. As predicted by simulations, a decrease in droplet diameter was observed when the water flow rate of the first nozzle was increased (Fig. 4c). At the same time, the frequency of droplet generation increased due to the constant oil flow rate. Furthermore, an increase of droplet diameter with increasing oil flowrate at a constant water flow rate was observed, analog to the simulation predictions (Fig. 4d). While the CFD model captured the general trends of droplet generation well, the droplet volumes are overestimated by 25% by the model. The cause for this can be found in the assumptions made in the construction of the model. Firstly, the channel walls were only characterized in their wetting behavior for water and not for mineral oil. Secondly, the level set method is based on reinitialization techniques which greatly affect accuracy and efficiency. Combined with the known mass loss problems of the method, this leads to the observed deviations [33]. The deviation could be removed by introducing an experimentally determined correction factor into the model. However, if the model is used for design purposes, this might not be required. Lastly, the performance of the second nozzle was tested and compared to simulation results. As predicted by simulations, a linear relationship between the water injection rate at the second nozzle and the distance between the droplets was found. By increasing the distance between the droplets aggregation and merging of droplets could be prevent (Fig. 4e). While in the case of using only a single nozzle, many of the droplets aggregated and merged at the outlet of the microfluidic device, the dual-nozzle microfluidic device effectively prevented this problem, allowing the dispensing of droplets as they were generated in the microfluidic device (Additional file 1: Figure S1).

## Conclusion

Here, we have shown a microfluidic device for the generation and dispensing of droplets. The microfluidic device consists of two separate nozzles. While the first nozzle is used for the generation of droplets, the distance between the individual droplets can be adjusted using the second nozzle. Using this method, the agglomeration and merging of droplets at the microfluidic device outlet can be prevented and dispensing of homogenous droplets can be achieved. The microfluidic device could be a valuable tool for a wide range of applications. Through the small size of the device, it might be particularly interesting for point-of-care applications.

## Additional file

**Additional file 1: Figure S1.** Microscope images of droplet dispensing from the microfluidic droplet dispenser for the case of a single nozzle (A) and dual-nozzle microfluidic device (B). Scale bars are 200µm. The droplets aggregate once they leave the microfluidic device when a single nozzle is used (Bottom section of both images show the dispensed droplets), while no aggregation or merging can be observed in the case of the dual-nozzle microfluidic device.
