# Supplementary material for: Dual-nozzle microfluidic droplet generator
Source: Nano Converg. 2018 May 8;5:12. doi: 10.1186/s40580-018-0145-2 (PMC5938299; doi:10.1186/s40580-018-0145-2)
Supplement: Supplementary file 1 — Additional file 1: Figure S1. Microscope images of droplet dispensing from the microfluidic droplet dispenser for the case of a single nozzle (A) and dual-nozzle microfluidic device (B). Scale bars are 200µm. The droplets aggregate once they leave the microfluidic device when a single nozzle is used (Bottom section of both images show the dispensed droplets), while no aggregation or merging can be observed in the case of the dual-nozzle microfluidic device. [file 40580_2018_145_MOESM1_ESM.docx]

**Figure S1**

**
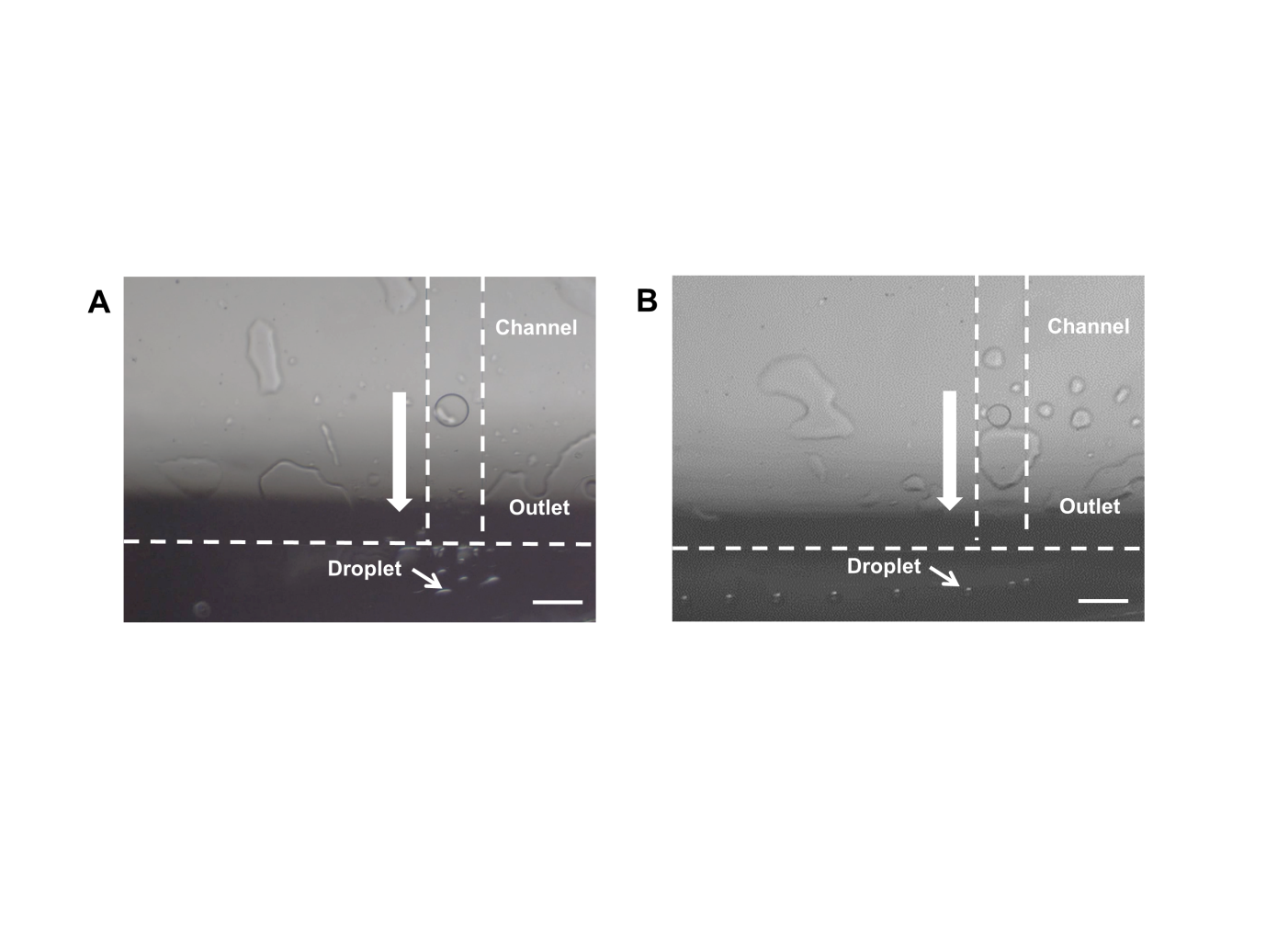
**

**Additional file 1: Figure S1.** Microscope images of droplet dispensing from the microfluidic droplet dispenser for the case of a single nozzle (A) and dual-nozzle microfluidic device (B). Scale bars are 200µm. The droplets aggregate once they leave the microfluidic device when a single nozzle is used (Bottom section of both images show the dispensed droplets), while no aggregation or merging can be observed in the case of the dual-nozzle microfluidic device.
